# Insulin resistance and inflammation mediate the association of abdominal obesity with colorectal cancer risk

**DOI:** 10.3389/fendo.2022.983160

**Published:** 2022-11-03

**Authors:** Wenqiang Li, Tong Liu, Liang Qian, Yiming Wang, Xiangming Ma, Liying Cao, Qingsong Zhang, Jun Qu

**Affiliations:** ^1^ Department of General Surgery, Aerospace Center Hospital, Beijing, China; ^2^ Department of Gastrointestinal Surgery/Clinical Nutrition, Capital Medical University Affiliated Beijing Shijitan Hospital, Beijing, China; ^3^ Department of Obstetrics and Gynecology, Hangzhou Women’s Hospital, Hangzhou, China; ^4^ Department of Hepatobiliary Surgery, Kailuan General Hospital, Tangshan, China; ^5^ Department of General Surgery, Kailuan General Hospital, Tangshan, China

**Keywords:** insulin resistance, inflammation, colorectal cancer, obesity, mediation

## Abstract

**Background:**

The close association of abdominal obesity rather than general obesity with colorectal cancer (CRC) risk might be mediated by IR and inflammation, which has never been systematically explored in large-scale prospective studies.

**Methods:**

We prospectively examined the mediation effects of the fasting triglyceride-glucose (TyG) index and C-reactive protein (CRP) on the associations of obesity (general and abdominal) with CRC risk among 93,659 participants. We used the Cox proportional hazards regression models and subgroup analyses to evaluate the hazard ratios (HRs) and 95% confidence intervals (95% CIs) of CRC. The CAUSALMED procedure was used to perform the mediation analyses.

**Results:**

During 13.02 years of follow-up, a total of 586 CRC cases were verified. Male participants with general obesity and abdominal obesity had a 1.29-fold and a 1.28-fold increased risk of CRC. However, a significant association was only observed among female individuals with abdominal obesity. Both TyG index and CRP were associated with an elevated risk of CRC, and A significant interaction between the TyG index and CRP was found for the risk of CRC (P for interaction<0.05). CRP and the TyG index significantly mediated the positive association between abdominal obesity and CRC risk.

**Conclusion:**

CRP and TyG index increased the risk of CRC independently and synergistically. Mediation effects of CRP and the TyG index were found for the association between abdominal obesity and CRC risk.

## Introduction

Colorectal cancer (CRC) is deadly and expensive to treat (1). Previous epidemiologic studies have reported a possible association between body size and the risk of CRC (2–6). Body mass index (BMI, in kg/m^2^) is positively correlated with CRC risk in men, while women have weaker correlations. In addition, abdominal obesity [as assessed by waist circumference (WC, in cm)] is closely associated with CRC risk in both sexes. One possible explanation for the disparity is that men and women have distinct body compositions, with fat constituting a more significant percentage of body mass in women (30%) than in men (20%) (7). Another explanation might be that abdominal obesity plays a crucial role in metabolic abnormalities, leading to chronic diseases, including cancer (8, 9).

The insulin resistance (IR) and inflammation hypotheses postulate that there is a relationship between abdominal obesity and CRC risk since the buildup of visceral fat is a significant predictor of IR and inflammation (10, 11). IR and inflammation have increased the risk of CRC incidence in experimental and epidemiologic investigations (12, 13). The fasting triglyceride-glucose (TyG) index is a simple and cost-effective way to detect IR (14) compared to the gold standard hyperinsulinemic-euglycemic glucose clamp approach (15). High-sensitivity C-reactive protein (hs-CRP), also known as CRP assessed by high-sensitivity assays, is a typical protein produced in response to inflammation, infection, and tissue damage that has been linked to chronic disorders such as cardiovascular disease (CVD) and cancer (16, 17). Based on the evidence above, we assume that the close association of abdominal obesity rather than general obesity with CRC risk might be mediated by IR and inflammation, which, to our knowledge, has never been systematically explored in large-scale prospective studies.

The Kailuan study is an ongoing, prospective cohort study that includes biennial follow-ups for each participant. The anthropometric and laboratory parameter measurements offer us a valuable opportunity to investigate 1) the association of the TyG index and CRP with the risk of CRC incidence and 2) the mediation effects of the TyG index and CRP on the associations of obesity (general and abdominal) with CRC risk.

## Methods

### Study population

As previously stated (17), this study was based on the Kailuan Study, a community-based ongoing cohort study performed in Tangshan city. The current study investigated the risk factors for chronic diseases such as cancer. In short, a sum of 101,510 individuals including 81,110 males and 20,400 females underwent a standardized questionnaire, physical examination, and laboratory testing from June 2006 to October 2007 (baseline). Follow-up examinations were carried out biennially to keep participants up to update participant status on the parameters above.

**Figure 1:**
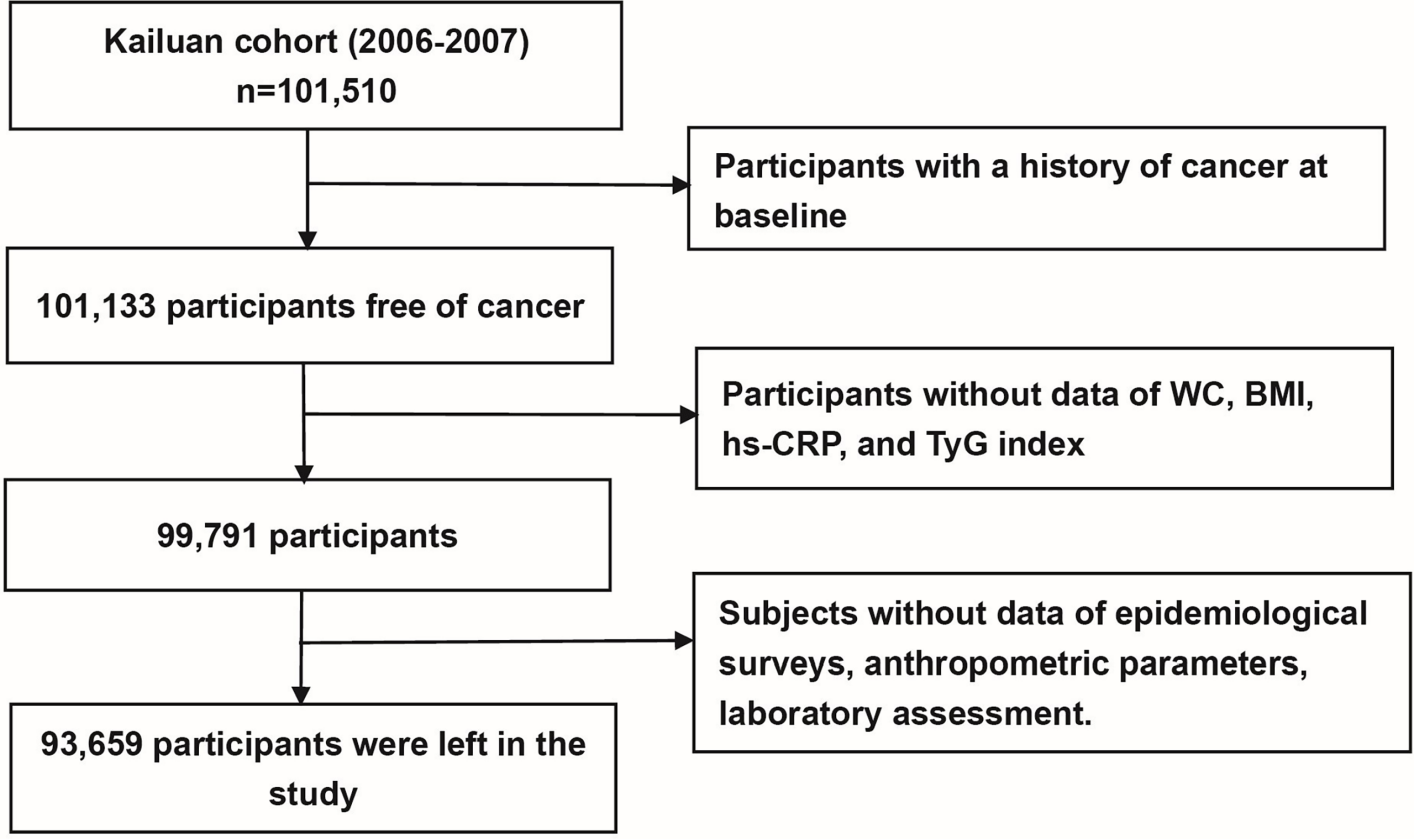
Flow chart of study participants.

In this study, individuals were excluded if they 1) were diagnosed with cancer previously(n=377); 2) had missing data on BMI, WC, CRP and the components of the TyG index, including fasting blood glucose (FBG, in mmol/L) and triglycerides (TG, in mmol/L) (n=1,342); and 3) lacked information about any potential confounders, including age, sex, social economic factors, laboratory tests and lifestyle behaviors (n=6,132). After factoring for the exclusion criteria, 93,659 individuals were enrolled, including 18,988 women and 74,671 men (**Figure 1**).

### Laboratory assessments

Patient’s venous blood was drawn into EDTA tubes after an overnight fast (8–12 h). All blood samples were analyzed in the Central Laboratory of Kailuan General Hospital using an autoanalyzer (Hitachi 747; Hitachi). The details regarding the assessment of plasma CRP, FBG, HDL-C, TG, and TC can be found elsewhere (18). The TyG index was estimated using the following calculations: ln [TG (mg/dL) × FBG (mg/dL)/2]. According to the Centers for Disease Control and Prevention and the American Heart Association guidelines, low-grade inflammation was defined as CRP ≥ 3 mg/L (19). The median of the TyG index (8.59) was used as the cutoff for the definition of IR.

### Ascertaining the outcome

For the duration of the follow-up, incident CRC cases were identified via: 1) tracking participants’ biennial health check-ups; 2) examining medical records linked with the Tangshan medical insurance system and the Kailuan Social Security Information System once a year, and 3) checking death certificates from the Provincial Vital Statistics Offices (PVSO) to further confirmed the outcome yearly. Clinical professionals assessed medical records and pathology reports to reconfirm the diagnosis of incident CRC, and CRC patients were categorized as C18-21 using the International Classification of Diseases, Tenth Revision (ICD-10).

### Potential confounders

Information on age, sex, socioeconomic factors, living habits, and personal and family members’ medical histories was collected *via* a standard questionnaire. BMI was calculated as body weight divided by the square of the body height and was grouped into the following three categories: normal (< 24.0 kg/m^2^), overweight (24.0-27.9 kg/m^2^), and general obesity (≥ 28.0 kg/m^2^). Abdominal obesity was defined as WC >90 cm in men and >85 cm in women. Drinking was defined as consuming ≥ 100 mL/day of alcoholic beverages for more than 6 months. Smoking status was defined as consuming ≥ 1 cigarette/day for more than 6 months. Physical activity was defined as having ≥ 3 times weekly with each time lasting ≥ 30 minutes based on the response to the question of frequency of Physical activity. Tea consumption was defined as ≥ 5 times weekly regardless of the tea types. High-fat diets were evaluated in the questionnaire as “Regularly” consumed.

### Statistical analysis

The mean ± SD and T-test were used to describe and compare continuous variables in the normal distribution. The median (IQR) and nonparametric tests (Kruskal-Wallis test) were used to describe and compare the skewed distributed variables (e.g., CRP and TG). Absolute values with percentages and the chi-square test were utilized to represent and compare categorical variables. Person-years were computed from the date of the baseline examination to the date of CRC diagnosis, death, or December 31, 2019, whichever occurred first. The hazard ratios (HRs) and 95% confidence intervals (CIs) for the development of incident CRC were calculated using Cox proportional hazards models. We firstly explored the association of general obesity (assessed by BMI) and central obesity (assessed by WC) with subsequent CRC risk among men and women, due to the distinct body compositions between men and women. Secondly, we investigated the effect of IR (assessed by TyG index) and inflammation (assessed by CRP) on the occurrence of CRC, multiplicative models were used to examine the interactions between CRP, TyG index, and CRC risks. Third, because there was an interaction between TyG index, CRP, and CRC risk, participants were further divided into four groups based on the presence/absence of the elevated TyG index (≥ 8.59) and CRP (>1 mg/L).

The CAUSALMED procedure was used to perform the mediation analyses based on the variance-covariance matrix and the maximum likelihood method. It calculated the total effect (the total of the direct and indirect effects), the direct effect (the effect without the mediator’s influence), and the indirect effect (the effect of the independent variable on the mediator multiplied the effect of the mediator on the outcome). All *P* values < 0.05 (two-sided) were judged statistically significant. Statistical analyses were carried out using SAS software (SAS Institute, Cary, NC, USA), version 9.4.

## Results

The baseline characteristics of the participants stratified by sex are listed in **Table 1**. The average age of the study population was 51.48 ± 12.44 years. Significant age differences, and levels of FBG, HDL-C, TC, TG, CRP, BMI, and WC, were found across the different sex groups. In addition, the percentages of the male sex, reported income, marital status, educational levels, physical exercise, tobacco, alcohol and tea consumption, sedentary lifestyle, high-fat diets, hypertension, diabetes mellitus, and family history of cancer differed considerably across the two groups.

**Table 1:** Baseline characteristics of the participants stratified by sex.

Variables	Overall	Women	Men	*P*-value
**n**	93,659	18,988	74,671	
**Age (year)**	51.44 ± 12.45	48.67 ± 11.46	52.15 ± 12.60	<0.001
**FBG (mmol/L)**	5.48 ± 1.68	5.32 ± 1.64	5.52 ± 1.69	<0.001
**HDL-C (mmol/L)**	1.55 ± 0.40	1.59 ± 0.39	1.54 ± 0.40	<0.001
**TG (mmol/L)**	1.27 (0.90,1.93)	1.18 (0.82,1.75)	1.30 (0.92,1.98)	<0.001
**TC (mmol/L)**	4.95 ± 1.15	4.98 ± 1.09	4.94 ± 1.16	<0.001
**CRP (mg/L)**	0.80 (0.30,2.06)	0.80 (0.30,2.28)	0.80 (0.30,2.00)	0.002
**BMI (Kg/m^2^)**	25.05 ± 3.50	24.66 ± 3.81	25.15 ± 3.41	<0.001
**WC (cm)**	86.90 ± 9.99	82.89 ± 10.70	87.92 ± 9.53	<0.001
**Per capita income (>800 ¥)**	13412 (14.32)	2984 (15.72)	10428 (13.97)	<0.001
**Educational background (High school or above, %)**	18698 (19.96)	5487 (28.90)	13211 (17.69)	<0.001
**Physical exercise (yes, %)**	14648 (15.64)	2545 (13.40)	12103 (16.21)	<0.001
**Current smoker (%)**	28948 (30.91)	268 (1.41)	28680 (38.41)	<0.001
**Current drinker (%)**	16760 (17.89)	94 (0.50)	16666 (22.32)	<0.001
**Family history of cancer (yes, %)**	3428 (3.66)	867 (4.57)	2561 (3.43)	<0.001
**Diabetes mellitus (yes, %)**	8501 (9.08)	1480 (7.79)	7021 (9.40)	<0.001
**Hypertension (yes, %)**	40861 (43.63)	6075 (31.99)	34786 (46.59)	<0.001
**Tea consumption (yes, %)**	8818 (9.42)	760 (4.00)	8058 (10.79)	<0.001
**Sedentary lifestyle (> 8 h/d, %)**	3038 (3.24)	710 (3.74)	2328 (3.12)	<0.001
**High-fat diets (regularly, %)**	8626 (9.21)	938 (4.94)	7688 (10.30)	<0.001

CRP, high-sensitivity C-reactive protein; BMI, body mass index; FBG, fasting blood glucose; HDL-C, high-density lipoprotein cholesterol; TG, triglyceride; WC, waist circumference; TC, total cholesterol.

During 13 years of follow-up, a total of 586 incident CRC was developed. **Table 2** shows the association of general obesity or abdominal obesity with CRC risk. Among the male group, participants with general obesity and abdominal obesity had a 1.29-fold (HR [95%] CI: 1.29, 1.01-1.64) and a 1.28-fold (HR [95%] CI: 1.28, 1.07-1.52) increased risk of CRC. However, a significant association was only observed among female individuals with abdominal obesity (WC >_85.0 vs. ≤85.0_, HR [95%] CI: 1.22, 1.03-1.50).

**Table 2:** The association of general obesity or central obesity with CRC risk.

	Men			Women		
	Case/person-years	HR (95%CI)	p-value	Case/person-years	HR (95%CI)	p-value
**General obesity^a^**						
** Normal**	174/339508	Ref.		32/111878	Ref.	
** Overweight**	223/396041	1.10 (0.90,1.34)	0.368	34/84586	1.05 (0.65,1.72)	0.835
** Obesity**	108/172796	1.29 (1.01,1.64)	0.040	15/41100	0.89 (0.48,1.67)	0.725
**Central obesity^b^**						
** No**	281/586301	Ref.		41/151814	Ref.	
** Yes**	224/322045	1.28 (1.07,1.52)	0.008	40/85750	1.22 (1.03,1.50)	0.013

Adjustments were made for age (every 10 years), family income, educational background, marital status, TC, smoking status, drinking status, physical activity, sedentary lifestyle, tea consumption, salt intake, high-fat diet, hypertension, and family history of cancer.

^a^General obesity was defined as BMI≥28.0 Kg/m^2^, and overweight was defined as BMI with a range of 24.0-27.9 Kg/m^2^.

^b^Central obesity was defined as WC> 90.0 cm for men, and WC> 85.0 cm for women.

**Figure 2:**
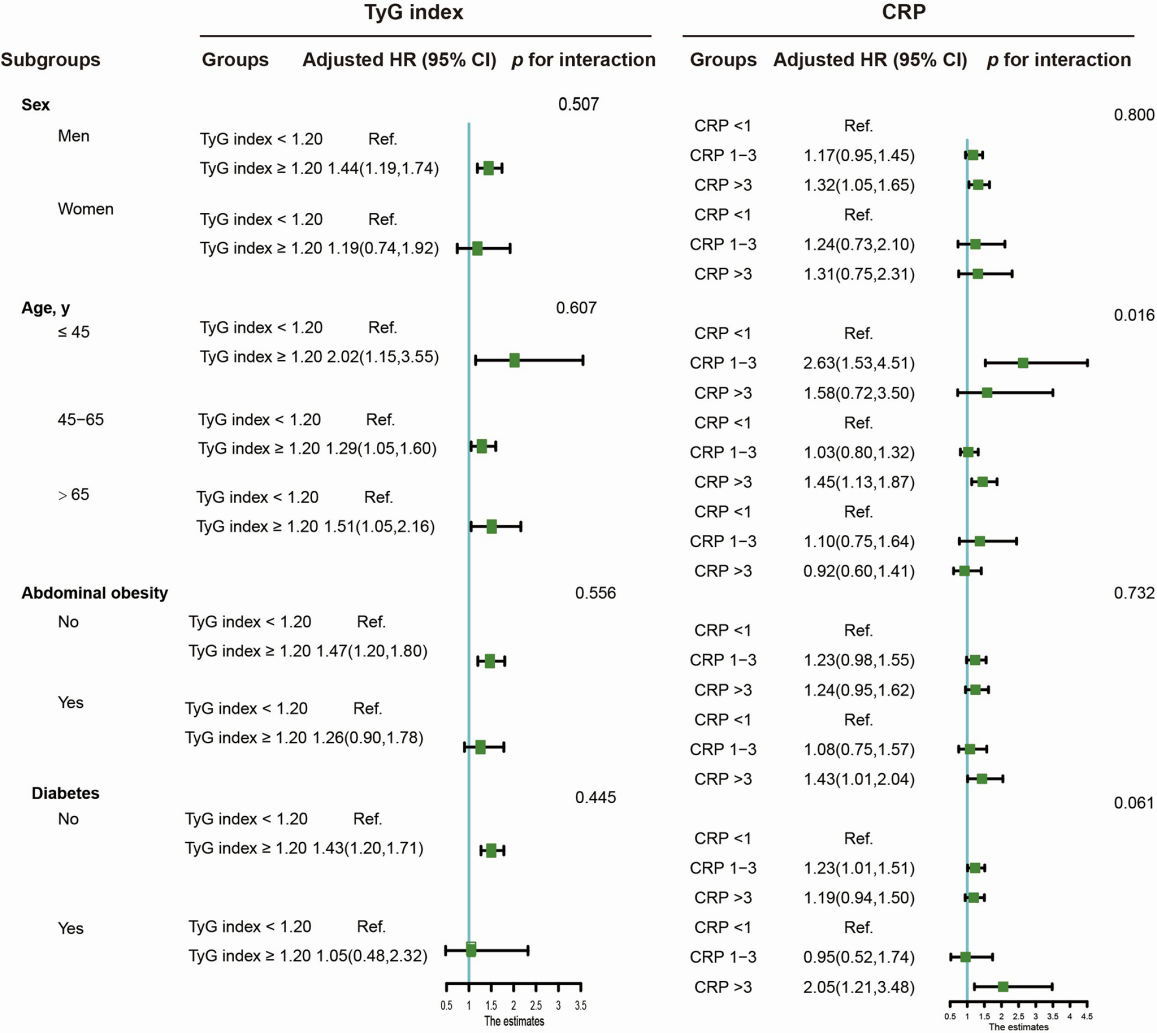
Subgroup analysis of the TyG index or CRP level association with CRC risk. Adjusted models were adjusted for age (every 10 years), sex, family income, educational background, marital status, WC, TC, smoking status, drinking status, physical activity, sedentary lifestyle, tea consumption, salt intake, high fat diet, hypertension, and family history of cancer.

The adjusted HRs (95% CI) for the association of the TyG index and CRP with the risk of CRC are shown in **Table 3**. TyG index (continuous) and elevated TyG index (≥ 8.59 vs. <8.59) were positively related to the risk of CRC incidence, with corresponding HRs (95% CI) of 1.21 (1.06-1.37) and 1.41 (1.17, 1.67), respectively. A significant interaction between the TyG index and inflammation (CRP> 3 mg/L) was found for the risk of CRC (*P* for interaction<0.05). There was a statistically significant trend of increasing relative risks of CRC across different CRP groups (CRP >_3 vs. <1_, HR [95%] CI: 1.29, 1.05-1.60; p for trend=0.042). **Figure 2** illustrates the subgroup analyses stratified by sex, age, abdominal obesity, and diabetes. Significant associations of an elevated TyG index with CRC risk were found among participants who were male, young, middle-aged, elderly, and without abdominal obesity or diabetes. Age significantly modified the associations between CRP and CRC risk (P for interaction < 0.05). The associations were more pronounced among young participants than middle-aged and elderly adults. The positive results were also observed when participants were stratified by sex, abdominal obesity and diabetes.

**Table 3:** Hazard ratios (HRs) for the association of TyG index or CRP levels with CRC risk.

Group	Cases/person-years	Crude models		Adjusted models	
		HR (95%CI)	*p*-value	HR (95%CI)	*p*-value
**TyG index (continuous)**	586/1145910	1.32 (1.17,1.47)	<0.001	1.21 (1.06,1.37)	0.006
**TyG index (median)**					
** < 8.59**	228/572835	Ref.		Ref.	
** ≥ 8.59**	358/573075	1.57 (1.33,1.84)	<0.001	1.41 (1.17,1.67)	<0.001
***P* for interaction^a^**					0.048
**CRP (continuous)**	586/1145910	1.01 (1.00,1.02)	0.128	1.00 (0.99,1.01)	0.758
**CRP**					
** < 1 mg/L**	291/672247	Ref.		Ref.	
** 1-3 mg/L**	162/277672	1.36 (1.12,1.65)	0.002	1.17 (0.97,1.43)	0.109
**>3 mg/L**	133/195992	1.59 (1.29,1.95)	<0.001	1.29 (1.05,1.60)	0.017
***P* for trend**			<0.001		0.042

Adjusted models were adjusted for age (every 10 years), sex, family income, educational background, WC, TC, smoking status, drinking status, physical activity, sedentary lifestyle, tea consumption, high-fat diet, hypertension, diabetes, and family history of cancer.

^a^Interaction between TyG index and CRP for the risk of CRC.

The significant interaction between the TyG index and inflammation affects CRC development. We further divided participants into four groups based on the absence/presence of an elevated TyG index and CRP (**Table 4**). After adjustments were made for the potential confounders, compared with the low TyG index and CRP group, participants with only an elevated TyG index or with an elevated TyG index and CRP had a 1.41-fold (HR [95%] CI: 1.41, 1.16-1.72) and 1.74-fold (HR [95%] CI: 1.74, 1.31-2.28) elevated risk of CRC.

**Table 4:** Hazard ratios (HRs) for the association of TyG index and CRP levels with CRC risk.

Group	Cases/person-years	Crude		Adjusted	
		HR (95%CI)	*p*-value	HR (95%CI)	*p*-value
**TyG(-) CRP(-) group**	179/482505	Ref.		Ref.	
**TyG(-) CRP(+) group**	49/90337	1.47(1.07,2.02)	0.017	1.25(0.91,1.72)	0.165
**TyG(+) CRP(-) group**	270/464199	1.57(1.29,1.90)	<0.001	1.42(1.17,1.73)	<0.001
**TyG(+) CRP(+) group**	88/108870	2.21(1.72,2.86)	<0.001	1.74(1.31,2.28)	<0.001

Adjusted models were adjusted for age (every 10 years), sex, family income, educational background, marital status, WC, TC, smoking status, drinking status, physical activity, sedentary lifestyle, tea consumption, high-fat diet, hypertension, and family history of cancer.

TyG (+): TyG index ≥ 8.59.

CRP (+): CRP ≥ 1 mg/L.

**Figure 3:**
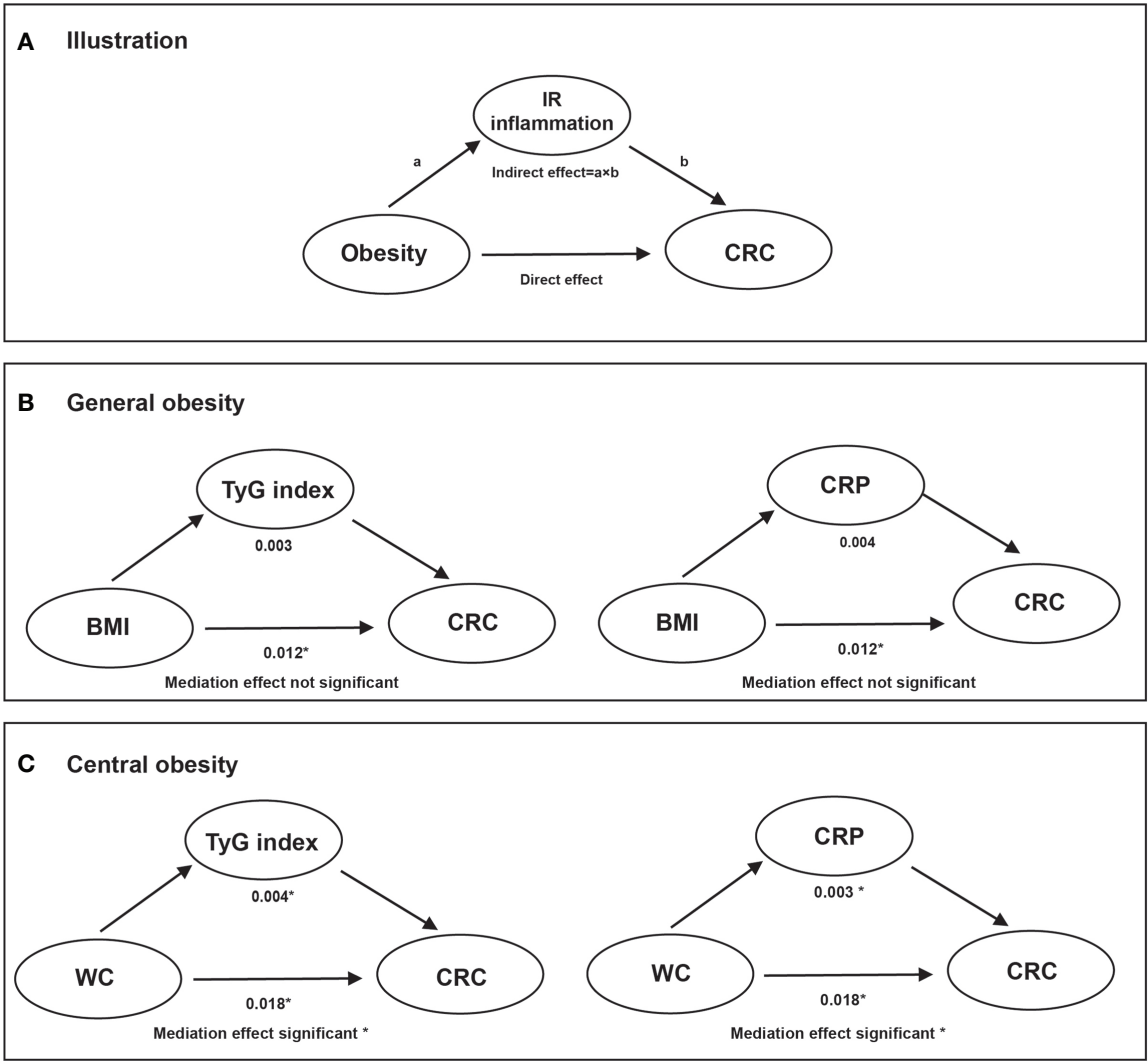
The mediation effect of TyG index and CRP on the association of obesity with CRC risk. Note: Adjusted models were adjusted for age (every 10 years), sex, family income, educational background, marital status, WC, TC, smoking status, drinking status, physical activity, sedentary lifestyle, tea consumption, salt intake, high fat diet, hypertension, and family history of cancer. **(A)**: illustration; **(B)**: overall obesity; **(C)**: central obesity. * Values were statistically significant.

In the mediation effect analysis, both CRP and the TyG index significantly mediated the positive association between abdominal obesity (elevated WC) and CRC risk after adjustments were made for the confounding factors. However, null or weaker mediation effects of the TyG index and CRP were found to associate general obesity with the risk of CRC incidence (**Figure 3**).

## Discussion

In this large-scale community-based cohort study, we found the following: I) abdominal obesity was associated with an elevated risk of CRC in both sexes, while general obesity was found to only increase the risk of CRC in men; II) TyG and CRP could raise the risk of CRC independently. In addition, IR along with inflammation may function synergistically to accelerate the initiation of CRC; III) CRP and TyG index only mediated the association between obesity assessed by WC and CRC risk, indicating the IR and inflammation hypotheses may help to explain the etiological importance of abdominal fat disposition, rather than overall adiposity.

We found that general obesity (BMI> 28 kg/m^2^) increased the risk of CRC among male participants, while abdominal obesity (WC>90 cm in men and >85 cm in women) was associated with an elevated risk of CRC incidence in both sexes. This finding is consistent with most previous epidemiological studies (2–6). The close associations between inflammation and CRC incidence have also been demonstrated in previous studies. A report from the general Danish population, which included 10,408 participants, found that elevated levels of CRP in cancer-free individuals were associated with an increased risk of cancer of any type and possibly CRC (20). A case-control study nested within the Japan Public Health Center-based prospective study found a 1.6-fold increased risk of subsequent colon cancer for the highest versus the lowest quartile of CRP (21). A systematic review including 5 nested case-control and 3 cohort studies identified a positive but weak association between pre-diagnostic circulating CRP concentrations and colorectal and colon cancer risk (12).

Little research has been designed to investigate the effect of the TyG index on the occurrence of CRC risk. In a retrospective population-based cohort study of 27,944 individuals, Takuro Okamura et al. found that the TyG index was a useful and accessible tool for predicting incident CRC. Recently, by analyzing 510,471 individuals from six European cohorts, Josef Fritz et al. found that the TyG index was associated with an increased risk of cancers of the digestive system, including colon, rectal, liver, and pancreatic cancer (22). Similarly, several epidemiological studies reported a close association between IR assessed by the homeostasis model assessment method (HOMA-IR) and the risk of colorectal cancer (23, 24), as did experimental studies (25, 26). A study speculated sulphonylureas may play a role in CRC carcinogenesis impairing the physiological insulin secretion among diabetes participants (27).

Epidemiological studies have found that WC is more significantly associated with CRC risk than BMI (28, 29), emphasizing the etiological importance of abdominal fat disposition rather than total adiposity. However, further direct evidence is needed to confirm this association. We found that CRP and the TyG index mediated the association of obesity, assessed by WC rather than BMI, with CRC risk. This finding partly explains the strong association between central obesity and CRC risk, that abdominal obesity-induced carcinogenesis may be through inflammatory and IR pathways. By using UK Biobank data, Dashti SG et al. exanimated the role of obesity-related factors including CRP, hemoglobin-A1c (HbA1c), sex hormone-binding globulin (SHBG), and testosterone in the association of adiposity and CRC risk (30). They found pathways influenced by CRP explained a small proportion of the adiposity-CRC association in both men and women. A prospective cohort study found that substantial proportions of the effect of BMI were mediated by the TyG index for cancers of the pancreas, rectum, colon, kidney, and liver. However, there were two limitations to the previous study. First, it did not explore the significance of those mediation effects. Second, WC was not assessed as an indicator of obesity. Abdominal obesity is a condition marked by low-grade chronic inflammation and IR. Adipose tissue functions as an endocrine organ, secreting a variety of proteins that regulate metabolism, energy intake, and fat storage, including leptin, adiponectin, interleukin- (IL-) 6, and tumor necrosis factor-alpha (TNF-α) (31).

The underlying mechanism by which inflammation and IR increase subsequent CRC risk includes two aspects. First, long-term, low-grade inflammation, which causes protein and DNA damage, can increase tumor growth and progression. Critical pathways that maintain normal cellular homeostasis can be altered by genetic and epigenetic variations in response to inflammatory mediators such as cytokines, free radicals, prostaglandins, and growth factors. Point mutations in tumor suppressor genes, DNA methylation, and posttranslational modifications are examples of these alterations, all of which can contribute to the existence and development of cancer (32). Second, insulin promotes colon cancer progression by increasing the expression of acyl-coenzyme A: cholesterol acyltransferase-1 (33), increasing the expression of vascular cell adhesion molecule-1 in intestinal tumor endothelial cells and causing a proinflammatory state (34), and elevating the levels of IGF-1, which promotes cell proliferation, survival, and angiogenesis by stimulating the synthesis of vascular endothelial growth factor (35).

The main strength of the current study is that it provides a unique perspective on the possible mediation effects of inflammation and IR on the association of obesity with future CRC risk based on a population-based cohort study. Additionally, this study fully considered the influence of potential confounders, such as lifestyle habits and a history of cancer-related illnesses. Additionally, the strengths of this study include the prospective study design, large sample size, and long-term follow-up. Sensitivity analysis and subgroup analyses were conducted to infer the robustness of our conclusion.

There are certain limitations in this study that should be mentioned. First, colon and rectal cancer could not be analyzed separately due to the scarcity of data. Inflammation and IR may have distinct carcinogenic impacts on the development of colon and rectal cancers. Second, although we controlled for most potential confounders, we could not exclude the possibility of residual cancer-related causal variables, such as the consumption of cereal, vegetable, and high-fiber foods, owing to a lack of information on how these products are consumed. On the other hand, dietary factors are substantially associated with BMI, TC, and TG. Because those factors were adjusted in the multivariate analysis, they may only have had a modest influence on the findings. Third, all participants were from the Kailuan community, with a higher proportion of men than women. As a result, this group could not be considered typical of the Chinese population. The findings could not be immediately extrapolated to other communities with various cultures and socioeconomic backgrounds. Fourth, instead of the gold standard, HOMA-IR, the TyG index was used as an indicator of insulin resistance, which may have resulted in misclassification and underestimation of the potential effect of IR.

## Conclusion

The results of this prospective cohort study showed that elevated CRP and TyG index increased the risk of CRC independently and synergistically. Mediation effects of CRP and the TyG index were found for the association between abdominal obesity and CRC risk, which may help to elucidate the etiological importance of abdominal fat disposition rather than overall adiposity.

## Data availability statement

The raw data supporting the conclusions of this article will be made available by the authors, without undue reservation.

## Ethics statement

The studies involving human participants were reviewed and approved by Aerospace Center Hospital and Kailuan General Hospital. The patients/participants provided their written informed consent to participate in this study.

## Author contributions

All authors have read and approved the manuscript. WQL: Methodology, Software, Writing-Original draft preparation. TL: Writing-Reviewing and Editing. LQ: Writing-Reviewing and Editing. YMW: Supervision, Validation. XMM: Software. LYC: Resources. QSZ: Conceptualization, Supervision. JQ: Conceptualization, Supervision, Validation, Resources. All authors contributed to the article and approved the submitted version.

## Acknowledgments

We thank all the staff and participants of the Kailuan study for their important contributions.

## Conflict of interest

The authors declare that the research was conducted in the absence of any commercial or financial relationships that could be construed as a potential conflict of interest.

## Publisher’s note

All claims expressed in this article are solely those of the authors and do not necessarily represent those of their affiliated organizations, or those of the publisher, the editors and the reviewers. Any product that may be evaluated in this article, or claim that may be made by its manufacturer, is not guaranteed or endorsed by the publisher.

## Abbreviations

BMI, body mass index; CIs, confidence intervals; CRC, colorectal cancer; CRP, C-reactive protein; CVD, cardiovascular disease; FBG, fasting blood glucose; HDL-C, high-density lipoprotein cholesterol; HOMA-IR, hyperinsulinemic-euglycemic glucose clamp; HRs: hazard ratios; TC, total cholesterol; TG, triglyceride; TyG, fasting triglyceride-glucose; WC, waist circumference.
